# Mitral Valve-in-Valve Implant of a Balloon-Expandable Valve Guided by 3-Dimensional Printing

**DOI:** 10.3389/fcvm.2022.894160

**Published:** 2022-05-30

**Authors:** Yu Mao, Yang Liu, Yanyan Ma, Ping Jin, Lanlan Li, Jian Yang

**Affiliations:** Department of Cardiovascular Surgery, Xijing Hospital, Fourth Military Medical University, Xi'an, China

**Keywords:** mitral valve, valve-in-valve, implant, balloon-expandable valve, 3-dimensional printing

## Abstract

**Background:**

Our goal was to explore the role of 3-dimensional (3D) printing in facilitating the outcome of a mitral valve-in-valve (V-in-V) implant of a balloon-expandable valve.

**Methods:**

From November 2020 to April 2021, 6 patients with degenerated mitral valves were treated by a transcatheter mitral V-in-V implant of a balloon-expandable valve. 3D printed mitral valve pre- and post-procedure models were prepared to facilitate the process by making individualized plans and evaluating the outcomes.

**Results:**

Each of the 6 patients was successfully implanted with a balloon-expandable valve. From post-procedural images and the 3D printed models, we could clearly observe the valve at the ideal position, with the proper shape and no regurgitation. 3D printed mitral valve models contributed to precise decisions, the avoidance of complications, and the valuation of outcomes.

**Conclusions:**

3D printing plays an important role in guiding the transcatheter mitral V-in-V implant of a balloon-expandable valve.

**Clinical Trial Registration:**

ClinicalTrials.gov Protocol Registration System (NCT02917980).

## Introduction

Transcatheter mitral valve implantation is developing as an alternative procedure for patients with a degenerated mitral valve and a high risk of severe postoperative dysfunction (1). The mitral V-in-V implant has been successfully used to treat degenerated valves (2–5). In addition, as 3D printing technology continues to develop, its application to various structural heart diseases has expanded (6, 7). With the rapid development of minimally invasive treatments in structural heart disease, clinicians have a greater need to know the exact cardiac anatomical structure. Compared with computed tomography (CT) scan, cardiovascular 3D printing is intuitive, personalized, and accurate, which may play an auxiliary guiding role in the treatment of these conditions (Table 1) (8, 9). Using preprocedural 3D printed models to help guide the transcatheter aortic valve replacement procedure has achieved positive results (10). However, there is minimal information focusing on the use of 3D printing to guide the mitral valve-in-valve (V-in-V) implant procedure. Our goal was to report on the use of 3D printing-guided transcatheter mitral V-in-V implants in 6 patients using the Prizvalve balloon-expandable valve (Newmed Medical Co., LTD., Shanghai, China).

**Table 1:** The advantages and disadvantages between 3D printing and CT scan.

	**3D printing**	**CT scan**
Advantages	- Visual spatial understanding - Hemodynamic assessment - Anticipating procedural complications - Optimal device selection - Physical properties similar to human structures - Bench test simulation for intervention - Communication with patients and trainees	- Fast scanning speed - High image resolution - Relatively clear images
Disadvantages	- Multi- discipline collaboration - Time- consuming - Cost	- Radiation - Need specialized analyzing software and staff - Lack of consistency of analyzing - Lack of physical characteristics

*3D printing, three- dimensional printing; CT, computed tomography*.

## Methods

### General Information

Table 2 lists the basic characteristics of the 6 patients who underwent surgical mitral valve procedures. There were 5 women and 1 man with a median age of 69 years (range 64–77 years). The mean duration from implanting the bioprosthesis implanted until it degenerated was 9 years (range 5–12 years). Overall, the patients had a high-risk profile, all of them had mitral regurgitation, and 2 patients had tricuspid regurgitation. Moreover, 2 patients had atrial fibrillation, and 2 patients had tricuspid insufficiency, which had hemodynamic implications. The median Society of Thoracic Surgeons score was 7.168% (range 5.110%−14.842%). Before the procedure, 4 patients were New York Heart Association functional class III and 2 patients were functional class IV. Patient 4 had previous coronary artery bypass grafting with patent grafts, and patient 6 had a pacemaker that had been implanted 5 years previously.

**Table 2:** Demographics of patients undergoing transcatheter mitral valve implantation.

**Patient number**	**Patient 1**	**Patient 2**	**Patient 3**	**Patient 4**	**Patient 5**	**Patient 6**
Age/Sex	75/F	77/F	72/F	66/M	66/F	64/F
Medical history	HT, AF, chole-cystectomy	HT, CHD, AF	HT, PHD	CHD, renal failure	Biological mitral valve degeneration	HT, diabetes
Previous heart operation (year)	MVR (9)	MVR (12)	MVR (11)	MVR+ CABG (11)	MVR+ TVR (6)	MVR+ pacemaker implanted (5)
Type of degenerated surgical bioprosthesis (Size)	Regent TM MHPJ-505 (29 mm)	Hall TM M7700 (27 mm)	Hall TM M7700 (27 mm)	Hall TM M7700 (27 mm)	Regent TM MHPJ-505 (29 mm)	Regent TM MEHPJ-505 (27 mm)
Current valve status	MR	MR, TR	MR	MR	MR, TR	MR
NYHA functional class	III	III	III	IV	III	IV
STS score (%)	5.110	7.415	6.063	14.842	6.921	12.166

*AF, atrial fibrillation; CABG, coronary artery bypass grafting; CHD, coronary heart disease; HT, hypertension; MVR, mitral valve replacement; PHD, pulmonary hypertension; STS, The Society of Thoracic Surgeons; MR, mitral regurgitation; TR, tricuspid regurgitation; Regent TM: St. Jude Medical Inc., Saint Paul, Minnesota, USA; Hall TM: Medtronic Inc., Minneapolis, Minnesota, USA*.

### Preprocedural Imaging Evaluation

**Figure 1:**
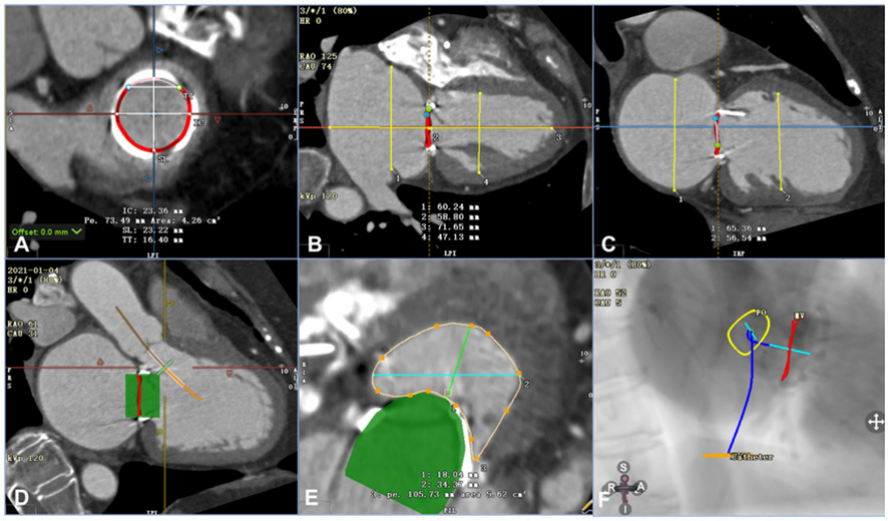
Assessment of computed tomography angiography before the procedure using Circle Cardiovascular Imaging CVI42 software (e.g., data for patient 6). **(A)** Annular area of the prosthetic mitral valve was 4.26 cm^2^. **(B)** The left ventricle was 71.65 mm in the long axis and 47.13 mm in the short axis, and the left atrium was 58.80 mm in the long axis and 60.24 mm in the short axis. **(C)** The left and right axes of the left ventricle and left atrium were 56.54 mm and 65.36 mm, respectively. **(D)** By simulating a 26-mm valve, the relationship between the stent and the neo-LVOT could be observed. **(E)** The area of the neo-LVOT was 5.62 cm^2^ after simulating the process of implanting the valve. **(F)** The projection angle of the released valve implanted by trans-septal access is RAO52, CAU5.

Transesophageal echocardiography (TEE) was performed on each patient before the procedure to measure and evaluate his or her anatomical abnormality. In addition, the patients' preprocedural computed tomography angiography (CTA) data were analyzed using Circle Cardiovascular Imaging CVI42 software (Calgary, Canada) (Figure 1). The inner and outer areas of the prosthetic mitral valve and the length and width of the left ventricle, the left atrium, the new left ventricular outflow tract area, and the projection angle were measured (Figure 1). Table 3 shows the preprocedural characteristics of the patients. The results showed that all the patients were suitable for the mitral V-in-V implant of the balloon-expandable valve.

**Table 3:** Preprocedural evaluations of patients undergoing transcatheter mitral valve implantation.

**Patient number**	**Patient 1**	**Patient 2**	**Patient 3**	**Patient 4**	**Patient 5**	**Patient 6**
Inner diameter of biological annulus (mm)	24.50	22.95	22.92	22.50	24.10	23.36
Height of biological valve (mm)	17.90	17.15	19.64	19.00	17.10	16.64
Left atrial diameter in diastole (long axis, short axis, left and right axes) (mm)	62.67, 68.73, 58.14	69.79, 71.34, 56.45	56.91, 65.02, 64.16	54.94, 59.42, 57.16	44.42, 47.74, 44.79	58.80, 60.24, 65.36
Left ventricle diameter in diastole (long axis, short axis, left and right axes) (mm)	69.57, 42.39, 40.18	70.63, 41.13, 42.12	76.36, 37.87, 39,59	70.43, 38.26, 38.93	81.87, 39.68, 51.69	71.65, 47.13, 56.54
Planned implanted valve size	29 mm	26 mm	26 mm	26 mm	26 mm	26 mm
Simulated LVOT area after valve implanted (cm^2^)	4.72	3.88	3.90	4.10	4.38	5.62

*LVOT, left ventricular outflow tract*.

### Device

**Figure 2:**
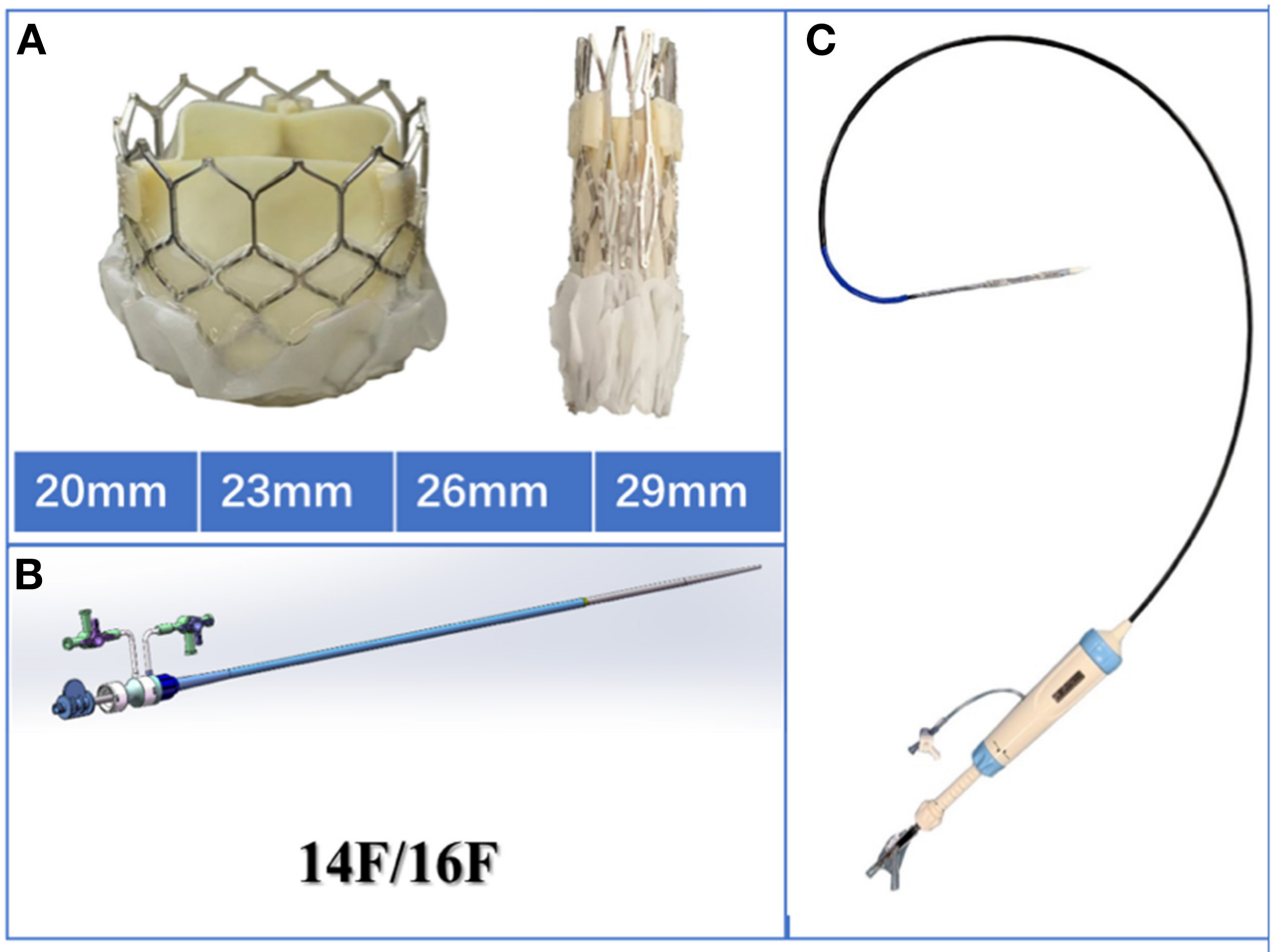
Characteristics of the Prizvalve balloon-expandable valve. **(A)** The 4 main sizes: 20, 23, 26, and 29 mm. **(B)** The 2 types of delivery systems (14/16F). **(C)** The adjustable curved introducer.

The Prizvalve balloon-expandable valve (Newmed Medical Co., LTD., Shanghai, China) (Figure 2), made of bovine pericardium, was used in the study. It is available in 4 sizes: 20 mm, 23 mm, 26 mm, and 29 mm. The bottom of the stent valve is covered with a polyester membrane, which can reduce the occurrence of paravalvular leakage. The delivery system is a double adjustable bendable sheath, which is more conducive to the coaxiality of the valve. There are three marked points in the middle of the valve that can be clearly seen under fluoroscopy to assist in the accurate positioning of the valve. The diameter of the outer sheath of the delivery system is 14/16 Fr, which meets the requirements of the peripheral blood vessels of most patients. At present, a clinical registration study of the Prizvalve is underway to enhance the information about the types of interventional valves independently developed in China and to provide significant treatment methods for older patients with severe valvular disease. The Prizvalve balloon-expandable valve is shown in Figure 2.

### Preprocedural 3D Printing Guidance

**Figure 3:**
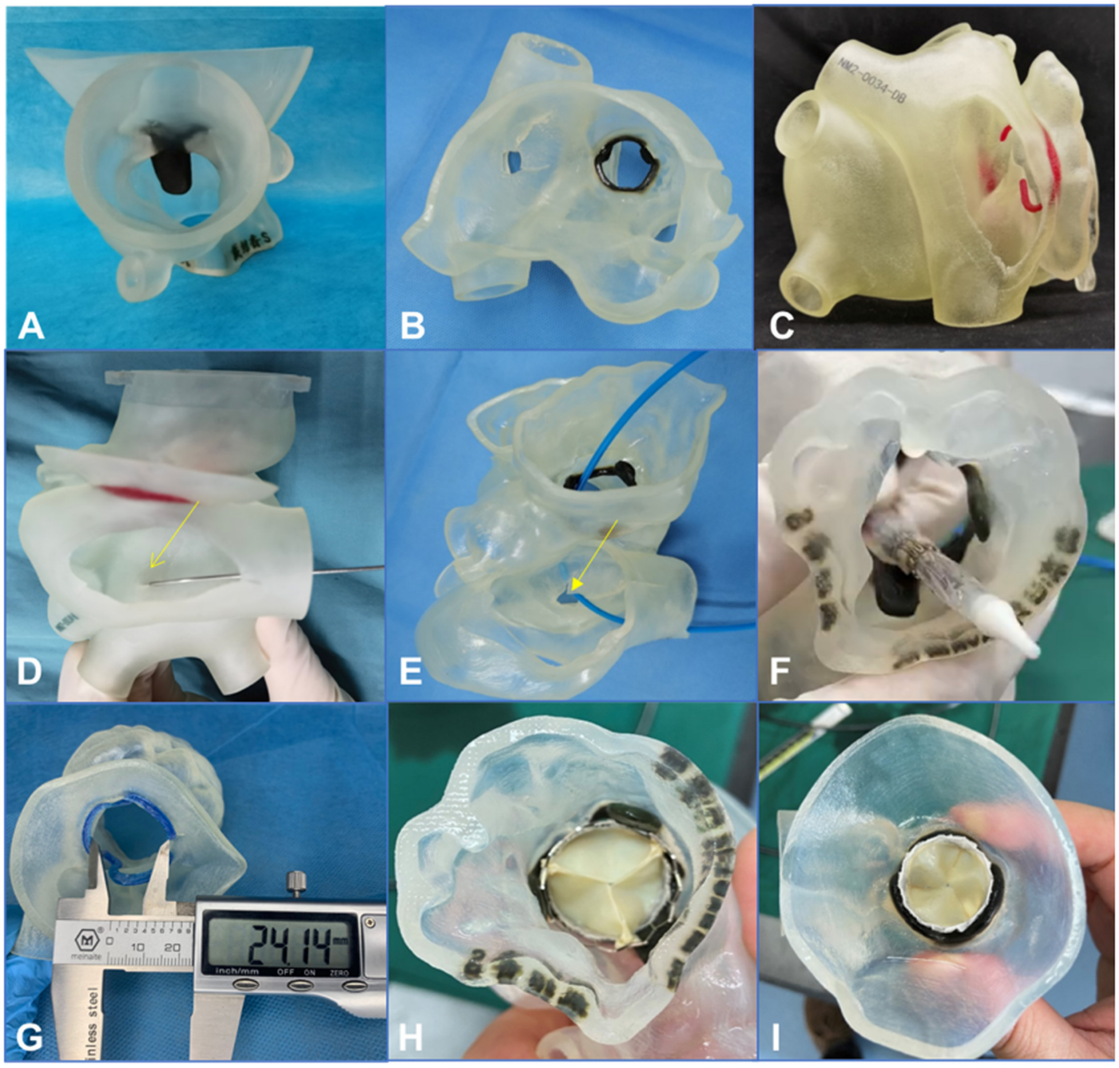
A 3-dimensional printed model was used to simulate the procedure in the bench test (e.g., data for patient 5). **(A–C)** 3-Dimensional printed model from the plane of the ascending aorta, the left atrium, and the right atrium, respectively. **(D–F)** An atrial septal puncture was simulated: The catheter went through the atrial septum and released the balloon-expandable valve successfully (the yellow arrows point to the puncture point). **(G)** The Vernier caliper was used to measure the inner diameter of the biological annulus. The result (24.14 mm) was equal to the result from the assessment made using computed tomography angiography. **(H,I)** Released valve from the plane of the left ventricular outflow tract and the left atrium.

The patients' CTA data were imported into Materialize Mimics 21.0 version software (Leuven, Belgium). Three orthogonal sections (coronal plane, sagittal plane, and cross plane) were established using the multiplane interactive reconstructed imaging function of the software based on actual needs. The mitral valve and the adjacent tissues were segmented by repeated comparisons and confirmations; then the 3D reconstruction and analysis of the delineated area were performed. The Materialize 3-Matic software was used to cut, smooth, repair, and extract the shell of the desired model. In addition, different parts of the tissues were distinguished by different colors to represent the multidimensional structure of each part such as the morphology, the distribution, and the interface. Finally, the 3D reconstructed model was exported into the Standard Tessellation Language format. The Standard Tessellation Language file was imported into a Polyjet 850 multimaterial full-color 3D printer (Stratasys, Inc., Eden Prairie, MN, USA), and different materials were selected for matching different tissues to print (Figures 3A–C). After going through a complex post-processing procedure, the 3D printed model was obtained for evaluation and simulation.

From the 3D printed model, we could better understand the specific anatomical structures of the mitral valve. In addition, the model could be used to simulate in the bench test when the trans-septal access was used, the goal being to test the effect of different locations of septal punctures on the coaxiality of the valve releases. The process of releasing of the balloon-expandable valve could also be simulated in the bench test so the procedure could be designed more accurately and effectively (Figures 3D-I).

### Procedure Strategy

The inner diameter of the annulus was about 23.39 ± 0.70 mm in the 6 patients. Which size to use was left to the discretion of the individual operator, informed by procedural TEE and the annular measurements and recommendations provided by the CT scans. When fully expanded, the 26-mm Prizvalve balloon-expandable valve was suitable for 5 patients (<15% oversize); the remaining patient was implanted with a 29-mm PrizValve balloon-expandable valve (<15% oversize).

Using the 3D printed models, we simulated the access procedures for the 6 patients. Five patients were suitable for trans-septal access, which was the least invasive approach. Patient 4 was not suitable for trans-septal access due to a sharp inferior vena cava–septum–left ventricular angle. The 3D model was simulated by atrial septum puncturing in the bench test, indicating that the catheter was relatively difficult to pass to the mitral valve from the left atrium. After a team discussion, we proposed that the procedure should be performed via the transapical access.

### Procedure

#### Trans-Septal Access

**Figure 4:**
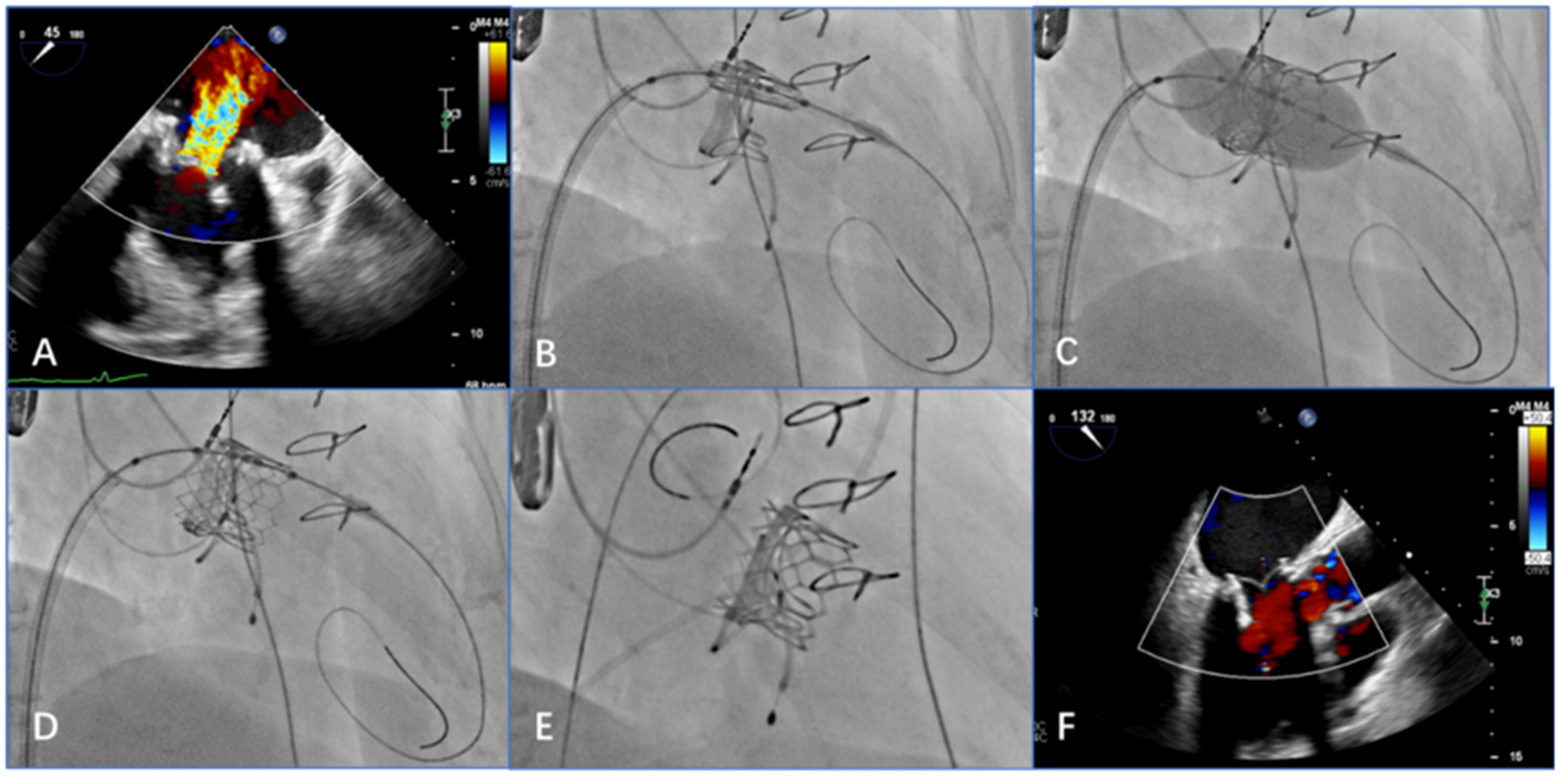
Transesophageal echocardiography (TEE) and digital subtraction angiography images show that the procedure achieved good results (e.g., data from patient 2). **(A)** Preprocedural TEE displays a large amount of colorful blood flow at the mitral valve. **(B)** The delivery system is positioned in relation to the mitral valve via the atrial septum. **(C)** After adjusting the position and the coaxiality, the balloon-expandable valve is inflated. **(D)** After expansion, the stent is fully unfolded. **(E)** When the guide wire is withdrawn, digital subtraction angiography shows that the position and shape of the mitral valve are ideal and that the stent fits closely to the valve. **(F)** Post-procedural TEE shows that the balloon-expandable valve is properly closed, with no paravalvular regurgitation.

Systemic heparinization to achieve an activated coagulation time of >200s, a 6F pigtail catheter was placed in the left ventricle through the left femoral artery sheath; the right femoral vein was punctured and imported slowly with a 16F sheath inserted into the inferior vena cava. The atrial septum was punctured under the guidance of TEE and fluoroscopy, and the atrial septum was fully expanded with a 14-mm balloon. The pigtail catheter was delivered along the guide wire to the left atrium, then across the mitral valve to the left ventricle. Left ventriculography showed massive mitral regurgitation (Figure 4A). The Prizvalve catheter delivery system loaded with the 26-/29-mm valve was advanced along the guide wire into the left ventricle, crossing the atrial septum and the bioprosthetic annulus frame, and the delivery system can be smoothly passed by the guidance of the guide wire. After the release position was determined, rapid ventricular pacing reached 180 beats/min, and the balloon stent was fully expanded. After procedures, the antithrombotic regimen included aspirin and clopidogrel, and Patient 2 was treated by warfarin.

#### Transapical Access

The transapical access was performed as previously described. Access to the left ventricular apex was gained through a 4- to 6-cm anterolateral minithoracotomy in the fifth or sixth intercostal space. The valve was loaded with a 26-mm Prizvalve catheter delivery system to the mitral valve by the guide wire. After the released position was determined, rapid ventricular pacing reached a rate of 180 beats/min. During rapid pacing, deployment was performed by inflating the balloon catheter. The exact positioning was guided by fluoroscopy and controlled by angiography. The antithrombotic regimen was performed as previously described.

Post-procedural TEE was used to examine the position and shape of the balloon-expanded valve and any regurgitation from the valve, which was consistent with the angiographic results (Figures 4B–F).

### Statistics

Continuous and normally distributed data are reported as mean ± standard deviation. A Student *t*-test was used for paired data testing. A *p* value of <0.05 indicated statistical significance. Statistical analyses were performed using SPSS 26.0 software (IBM Corp, Armonk, NY, USA).

## Results

### Procedure

**Figure 5:**
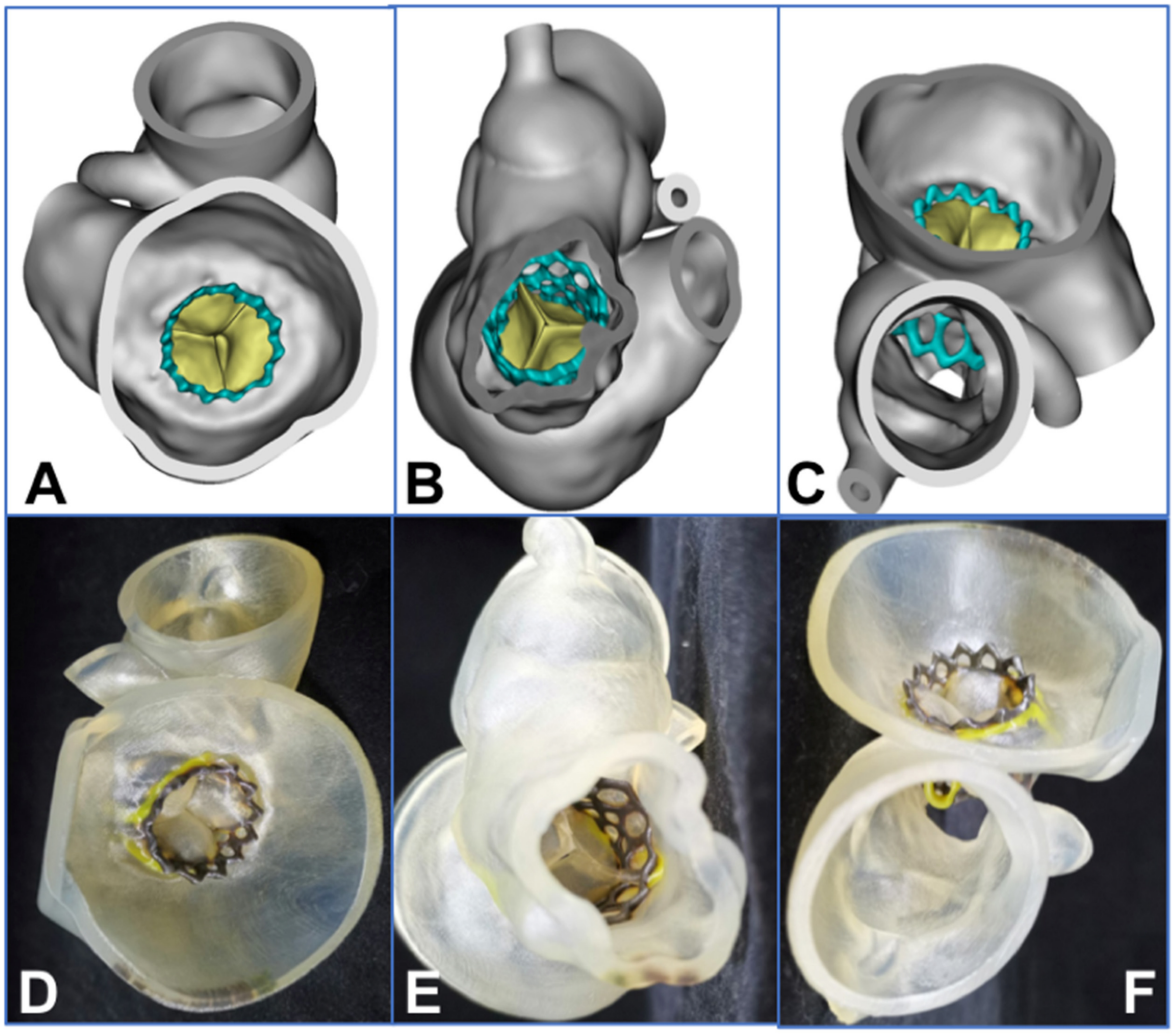
Assessment of 3-dimensional (3D) reconstruction is completed by using post-procedural computed tomographic data (e.g., data for patient 2). **(A–C)** 3D reconstruction from the plane of the left atrium, the left ventricle, and the ascending aorta, respectively. **(D–F)** The 3D printed model from the plane of the left atrium, the left ventricle, and the ascending aorta, respectively. The yellow area represents the frame of the degenerated valve, and the black area is the frame of the balloon-expandable valve.

The study enrolled 6 high-risk older patients who presented with multiple comorbidities. Transcatheter mitral V-in-V implants were technically successful in all patients, with no intraprocedural complications. Procedural characteristics are shown in Table 4. With the guidance of 3D printing, the procedural time and the use of digital subtraction angiography were obviously reduced. We used post-procedural CT data to complete the 3D reconstruction of the mitral valve and the adjacent tissues (Figure 5).

**Table 4:** Patients undergoing transcatheter mitral valve implantation using trans-septal access or transapical access.

**Patient number**	**Patient 1**	**Patient 2**	**Patient 3**	**Patient 4**	**Patient 5**	**Patient 6**
Left ventricular apex	Transfemoral	Transfemoral	Transfemoral	Transapical	Transfemoral	Transfemoral
Access	Trans-septal	Trans-septal	Trans-septal	Transapical	Trans-septal	Trans-septal
Closure of atrial septum		20 mm ASO				20 mm ASO
DSA time (min)	30	31	25	43	40	30
Procedure time (min)	120	145	100	115	135	130

*DSA, digital subtraction angiography*.

### Follow-Up Outcomes

**Figure 6:**
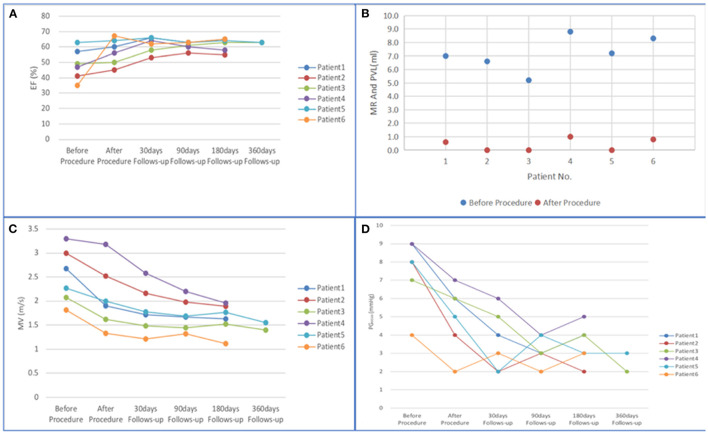
**(A–D)** Changes in mitral valve function from baseline to 360 days. (EF, ejection fraction; PVL, paravalvular leak; MR, mitral regurgitation; MV, maximal diastolic velocity through mitral valve; PG_MEAN_, mean diastolic gradient through mitral valve).

The follow-up results are shown in Figure 6 and Table 5. The ejection fraction increased significantly compared with preoperative values (48.67 ± 3.81 vs. 61.50 ± 1.91%, p < 0.01). Most patients showed no or mild paravalvular aortic regurgitation (100% [6 of 6]), confirmed by intraprocedural TEE. Moreover, a significant reduction in the mean diastolic gradient through the mitral valve from 7.50 ± 1.80 to 3.67 ± 1.49 mmHg was observed (*p* < 0.01). The maximal diastolic velocity through the mitral valve was reduced significantly compared with the preoperative values (2.52 ± 0.52 vs. 1.82 ± 0.44m/s, p < 0.05). At the 180-day follow-up, all patients presented with New York Heart Association functional class I (*n* = 4) or II (*n* = 2). Patient 4 had poor cardiac function on admission and presented with symptoms of heart failure and bleeding 13 h after the procedure. Patient 6 was transferred to the intensive care unit due to aspiration on the day after the procedure. The patient was resuscitated and discharged 14 days later. No rehospitalization was necessary during the follow-up period. As of 15 November 2021, no patients reported any adverse events. In addition, the median length of hospital stay was 9.5 days (range 4–17 days). The survival rate at 30 days was 100% (6/6).

**Table 5:** Follow-up of patients undergoing transcatheter mitral valve implantation.

**Patient number**	**Patient 1**	**Patient 2**	**Patient 3**	**Patient 4**	**Patient 5**	**Patient 6**
New Pacemaker implanted						
Complication before discharge				Heart failure, bleeding		Aspiration by mistake
Survival at 30 days	Yes	Yes	Yes	Yes	Yes	Yes
Length of hospital stay (days)	7	6	12	4	17	14

## Discussion

Currently, in addition to conventional surgery for valve replacement or valve repair, transcatheter mitral valve replacement and valve repair are available (11). For older patients undergoing mitral valve replacement with thoracotomy, the quality of the valve has certain limitations. If the biological valve degenerates within 10 years, the patient needs a second operation to replace the valve. But for the patients who have comorbidities, an extra operation would be a great risk. In recent years, with the continuous development of transcatheter valve replacement technology, transcatheter mitral valve implantation has developed rapidly (12, 13). TMVI in the setting of valve-in-valve has emerged as a promising strategy for failing bioprosthesis. Yoon SH. et al. displayed the outcomes of transcatheter mitral valve replacement for degenerated bioprostheses (14). Meanwhile, Simonato M. et al. has given the comprehensive midterm evaluation of valve-in-valve and valve-in-ring implantation from the VIVID registry (15). In addition, LVOT obstruction is a serious problem of great concern for TMVI. Russo G et al. showed the most common eligibility criteria and selection failures causes of left ventricle outflow tract (LVOT) obstruction (16). An area of 1.7 cm2 for the estimated neo-LVOT after TMVR has predicted LVOT obstruction accurately. Overall, due to complex anatomical structures of MV, TMVR still has several challenges for surgeons to overcome.

As material science and imaging technology continue to develop, the applications of 3D printing technology to the field of medicine have become more widespread. In recent years, 3D printing has played an important role in the fields of orthopedics, stomatology, and plastic surgery, to name a few (17, 18). In addition, cardiovascular diseases seriously endanger human health because of the inevitable surgical trauma from a traditional thoracotomy, and the surgical procedures take much longer than the minimally invasive procedures. 3D printing technology has been slower to develop in the field of cardiology because of the technical difficulties presented by the pulsatility and the elaborate, complex anatomical structures of the cardiovascular system. In addition, despite technological advances in the area of minimally invasive surgery, the operator cannot always clearly visualize the pathological changes in the anatomical structures of the patient because of the visual constraints imposed by the minimally invasive procedures. Cardiovascular 3D printing technology can display the anatomical structures in the different areas of heart individually, accurately, and intuitively. We believe that patients with valvular diseases with complex anatomical structures will benefit from the use of 3D printed models. From the preprocedural imaging data, we can evaluate the size and height of the annulus and the best projection angle of the released valve. Meanwhile, 3D printed models could be used to simulate the procedural process in the bench test, which has a certain auxiliary guiding role in the treatment of cardiovascular diseases to ensure the safety of the patient, the goal being to improve the surgical procedure and reduce the number of deaths (19, 20).

Cardiovascular 3D printing technology now plays a significant role in guiding transcatheter aortic valve replacement, transcatheter mitral valve replacement, transcatheter tricuspid valve replacement, and transcatheter pulmonary valve replacement (21–24). When one is contemplating using a thoracotomy to treat a complicated congenital heart disease, printing a 3D model of the patient's specific anatomical characteristics before the procedure enhances one's ability to plan the procedure. In addition, 3D printed models of patients with lack of room with special materials for the treatment of cardiovascular diseases, such as cardiomyopathy and left atrial appendage occlusion, could play an important role as well (25–27).

In the pre-procedural planning, the location of the transseptal puncture is important enough. Russo G et al. showed the procedural guidance and the challenging situations of the transseptal puncture (28). Preprocedural CT data were evaluated in detail and used to reconstruct the 3D model to help clinicians understand the specific anatomical structures and the appropriate puncture position on the interatrial septum in order to prepare personalized strategies for individual patients. Five patients were implanted with the 26-mm Prizvalve balloon-expandable valves by trans-septal access and patient 4 was implanted by transapical access. With the guidance of 3D printing, the procedures were technically successful in all patients. All 6 patients were followed up and recovered well without complications.

We combined 3D printing technology with a balloon-expandable valve for the first time in China, adopted the V-in-V technology to treat degenerated mitral valves, and achieved satisfactory clinical results.

### Limitations

The main limitation of the study is the small sample size. Multicenter and large-sample analyses are needed to evaluate the advantages of 3D printing. Also, this technique is expensive and time-consuming. Last but not least, more extensive mid- and long-term follow-up is needed to evaluate the safety and efficacy of the technique and the valve.

## Data Availability Statement

The original contributions presented in the study are included in the article/supplementary material, further inquiries can be directed to the corresponding author.

## Ethics Statement

The studies involving human participants were reviewed and approved by Clinicaltrials Organization: Xijing Hospital, Fourth Military Medical University. The patients/participants provided their written informed consent to participate in this study. Written informed consent was obtained from the individual(s) for the publication of any potentially identifiable images or data included in this article.

## Author Contributions

All authors listed have made a substantial, direct, and intellectual contribution to the work and approved it for publication.

## Funding

This work was supported by the National Key R&D Program of China (No. 2020YFC2008100), the Shaanxi Province Innovation Capability Support Plan—Innovative Talent Promotion Plan (No. 2020TD-034), and the Discipline Boosting Program of Xijing Hospital (No. XJZT18MJ69).

## Conflict of Interest

The authors declare that the research was conducted in the absence of any commercial or financial relationships that could be construed as a potential conflict of interest.

## Publisher's Note

All claims expressed in this article are solely those of the authors and do not necessarily represent those of their affiliated organizations, or those of the publisher, the editors and the reviewers. Any product that may be evaluated in this article, or claim that may be made by its manufacturer, is not guaranteed or endorsed by the publisher.
